# American Academy of Dental Science

**Published:** 1891-10

**Authors:** 

**Affiliations:** American Academy of Dental Science


### AMERICAN ACADEMY OF DENTAL SCIENCE.

The regular monthly meeting of the American Academy of Dental Science was held in the Boston Medical Library Association rooms on June 3, 1891, President Seabury in the chair.

The paper of the evening was read by Thomas Fillebrown, M.D., D.M.D. of Boston; subject, "Vitality as a Germicide."

(For Dr. Fillebrown's paper, see page 678.)

DISCUSSION.

*Dr. Potter.*—The most ardent advocate of antiseptic surgery would not undervalue the very great assistance rendered by the vitality of the tissues. Leucocytes have, without doubt, great germ-destroying power, but can we not in some way assist the vital power of the tissues?

To my mind there are several ways of so doing. First, by the mechanical removal of germs through washing. Secondly, by the drainage of wounds. Thirdly, by the use of substances which lessen the power and vitality of the germs, causing them to more readily yield to the germicidal action of the leucocytes; in other words, by an intelligent use of so-called antiseptics or germicides.

*Dr. Briggs.*—To any one who has given this subject thought, it must at times have seemed as though we had gone into this antiseptic treatment so thoroughly and with such enthusiasm that there would be a reaction, and that a time would come when with more knowledge much of our present treatment would appear to have been unwise or unnecessary. It does not seem to me that we have reached that point yet. Dr. Fillebrown speaks of the agencies which were developed at the time Listerism came into vogue. I have not looked into the subject, and I may not be correct in my statement, but my impression is in regard to the matter of skill that there has not been such great improvement. The improvement has been in the courage to do, because results were found to be better when aseptic conditions were produced. Listerism, as I understand it, was not so much asepticism as it was carbolicism. For a long time the only thing used was carbolic acid, as though that was the main thing and not the underlying principle of cleanliness. Nature makes things clean by washing and diluting, and it is understood that germs must be present in some certain force to produce certain results. I think I have seen the statement about a small-pox hospital that the air-space of six hundred feet was sufficient to prevent infection. Now, there must be some germs beyond that six hundred feet, but they do not get there in sufficient force to do damage. In our efforts at antisepticism we dilute and disperse these germs, even if we do not kill them all, so that they are not present in sufficient numbers to work injury.

To go back to the point spoken of about the skill, I think that we do the former operators great injustice when we speak so much about modern skill. The matter of drainage is not so important in the most advanced surgery of to-day. At the Massachusetts General Hospital they now take a patient with a compound fracture, and after having washed the parts and made everything clean, they put that leg up in a plastic splint, and let it alone till the fracture is united. Formerly, as you all know, a compound fracture was considered one of the most serious injuries, and drainage was thought absolutely necessary in treatment. So there is something (it may be the cleanliness) gained by the use of antiseptics. While I think we have overdone the antiseptic treatment, I do not think we should regard it as a past principle, and cast it aside. The use of antiseptics should go hand in hand with cleanliness. While antiseptics do not kill all germs, they do kill some, and inhibit others, and meanwhile the vitality of the patient carries on its work of repair.

*Dr. Williams.*—I have always understood that the use of antiseptics was simply as a road to, and, in many cases, a necessary way to arrive at, asepsis. We must have it in some way, and in some cases the leucocytes are not able to conquer the mischievous bacteria; then we must have some help, just as in many local and constitutional troubles we help nature to work in the way she wants to work. In our endeavors to get immunity from these morbid elements, whether we arrive at it by constitutional vigor or whether we help this vigor by neutralizing the enemy, we attain the same point. But we all know that constitutional strength is not always sufficient to overcome the enemy.

*Dr. Briggs.*—One word more. It is pretty hard to separate this absolute cleanliness from the use of antiseptics. I have not felt at any time that the use of strong germicides, so strong as possibly to be irritating to the parts, was necessary. Those are extreme ideas, but we do not have to go to the extremists to get the best methods, and it seems to me that if we wish to carry out the idea of making everything clean, cleanliness can be best obtained by the use of these antiseptics. We have got to use them on the instruments to destroy all possibilities of germ life, whether we give up the idea or not that a germicide must be used on the parts affected. If it goes forth that we can get along without antiseptics we are not going to get cleanliness, because our present means of sterilizing instruments is not sufficient unless we use those things.

William H. Potter, D.M.D.,
*Editor American Academy of Dental Science.*

